# Supplementary material for: Murine- and Human-Derived Autologous Organoid/Immune Cell Co-Cultures as Pre-Clinical Models of Pancreatic Ductal Adenocarcinoma
Source: Cancers (Basel). 2020 Dec 17;12(12):3816. doi: 10.3390/cancers12123816 (PMC7766822; doi:10.3390/cancers12123816)
Supplement: Supplementary file 1 [file cancers-12-03816-s001.pdf]

Supplementary Materials: Murine and Human-Derived Autologous Organoid/Immune Cell Co-Cultures as Preclinical Models of Pancreatic Ductal Adenocarcinoma

Loryn Holokai, Jayati Chakrabarti, Joanne Lundy, Daniel Croagh, Pritha Adhikary, Scott S. Richards, Chantal Woodson, Nina Steele, Robert Kuester, Aaron Scott, Mohammad Khreiss, Timothy Frankel, Juanita Merchant, Brendan J. Jenkins, Jiang Wang, Rachna T. Shroff, Syed A. Ahmad and Yana Zavros

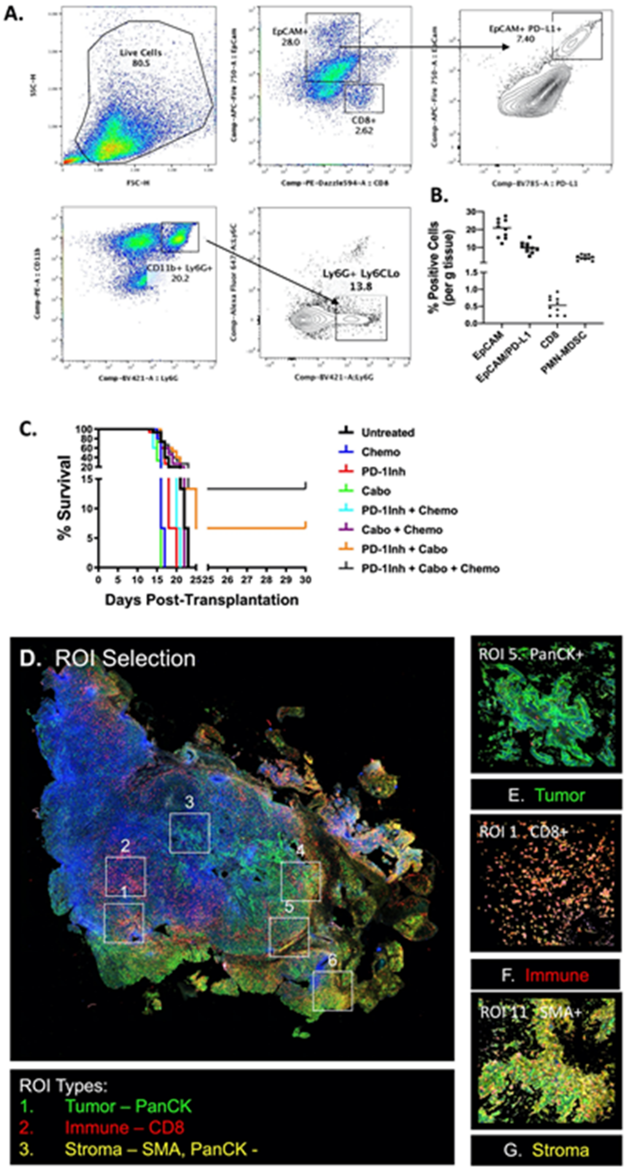

Figure S1. (A) Flow cytometric gating strategy for the quantification of EpCAM+/PD-L1+, CD8+, and CD11b+Ly6G+Ly6C- cell populations. **(B)** Quantification of major cell populations in gram per total tumor tissue. **(C)** Kaplan-Meier curve of survival in response to experimental treatment groups, n = 20 mice per group. Digital Spatial Profiling (DSP) demonstrating the **(D)** ROIs selection, and isolated **(E)** tumor, **(F)** immune, and **(G)** stroma compartment.

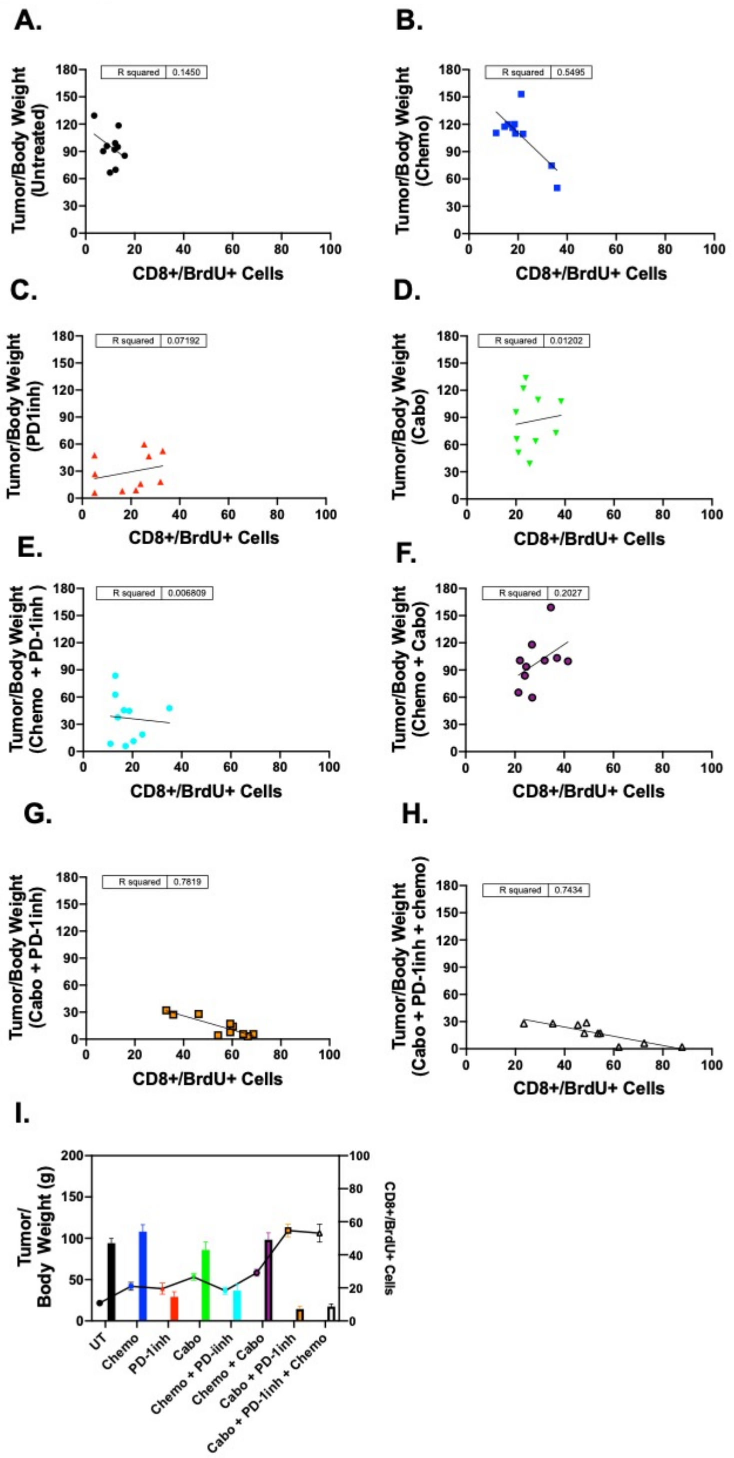

Figure S2. Regression analysis between tumor/body weight and CD8+/BrdU+ cells in different experimental groups: (**A**) untreated or treated with **(B)** Chemo **(C)** PD-1inh, **(D)** Cabo, **(E)** Chemo + PD-1inh, **(F)** Chemo + Cabo **(G)** Cabo + PD-1inh and **(H)** Cabo + PD-1inh + Chemo. **(I)**. Column-line graph showing the summarized correlation between tumor weight and CTL proliferation in each experimental group. *n* = 10 mice per group.

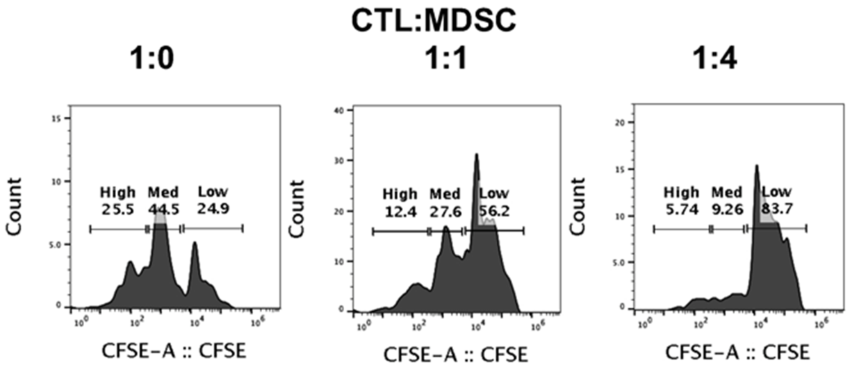

Figure S3. CFSE T cell proliferation assay in response to increased ratio of CTL: MDSCs.

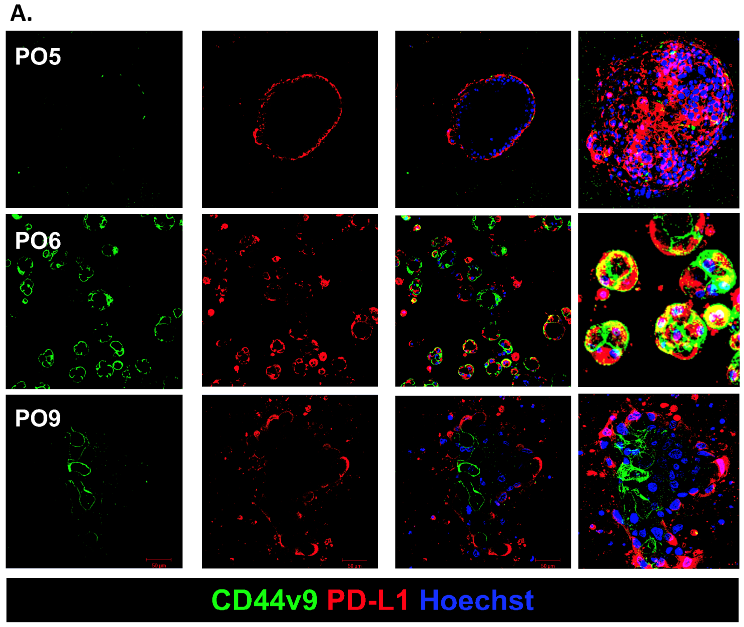

B.

Patient	Patient Chemotherapy Treatment	Stage
PO5	FOLFIRINOX (adjuvant)	pT3N1
PO6	FOLFIRINOX (adjuvant)	pT1N0
PO9	No treatment	pT3N1
PO10	Gemcitabine Abraxane	pT2N0
PO17	FOLFIRINOX (adjuvant)	pT1N0
PO24	FOLFIRINOX	pT3N1
PO26	FOLFIRINOX	pT2N2
PO28	Gemcitabine Abraxane	pT2N2
PO29	FOLFIRINOX (adjuvant)	pT2N1
PO30	FOLFIRINOX (adjuvant)	pT2N2

Figure S4. PD-L1 expression in organoids used for co-culture and patient information. **(A)** Immunofluorescence staining of patient-derived organoids for the expression of PD-L1 (red) and CD44v9 (green). **(B)** Table of patient tumor staging, tumor cell differentiation and invasion of adjacent tissue and lymph nodes.

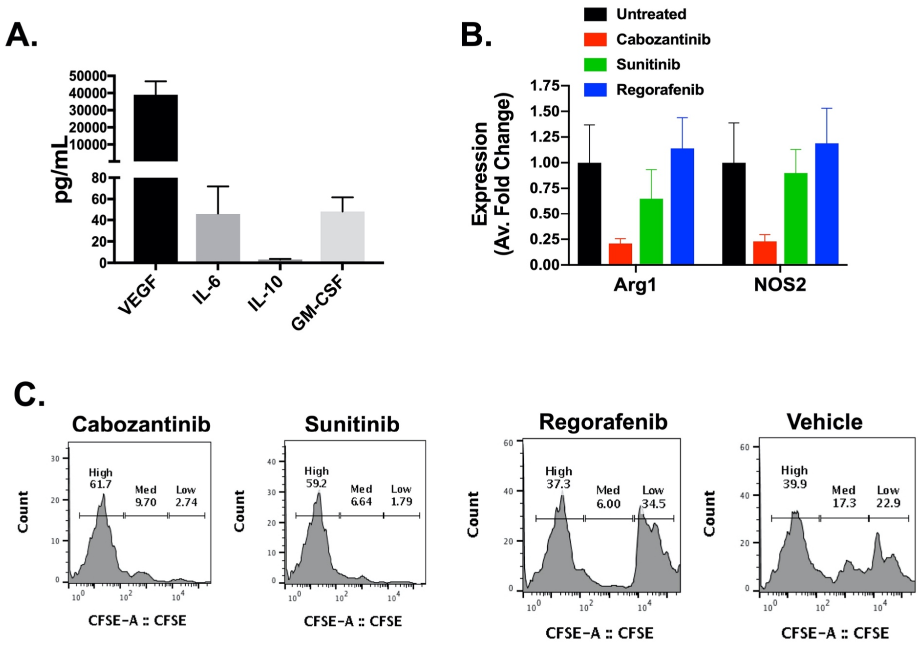

Figure S5. Immunosuppressive PMN-MDSC functional analysis. **(A)** Luminex assay of organoid conditioned media for the measurement of VEGF, IL-6, IL-10 and GM-CSF. Patient-derived MDSC were differentiated to PMN-MDSCs using cancer organoid conditioned media and co-cultured with CTLs. **(B)** Quantitative RT-PCR using RNA extracted from MDSC/CTL co-cultures treated with cabozantinib, sunitinib and regorafenib measuring Arg1 and NOS2 expression. **(C)** Changes in CTL proliferation as measured by CFSE T cell proliferation assay in MDSC/CTL co-cultures treated with cabozantinib, sunitinib and regorafenib was used to measure Arg1 and NOS2 expression. *P <0.05 compared to untreated group, n = 4 individual patient-derived PMN-MDSC/CTL co-cultures.

Publisher's Note: MDPI stays neutral with regard to jurisdictional claims in published maps and institutional affiliations.

© 2020 by the authors. Licensee MDPI, Basel, Switzerland. This article is an open access article distributed under the terms and conditions of the Creative Commons Attribution (CC BY) license (http://creativecommons.org/licenses/by/4.0/).
